# Niagara University

**Published:** 1888-05

**Authors:** 


### *NIAGARA UNIVERSITY.*

### CONVOCATION FOR CONFERRING DEGREES IN MEDICINE.

The annual convocation of Niagara University for conferring degrees, in connection with the work of its Medical Faculty, located in Buffalo, was held in the Association Hall on the evening of the 10th of April. The large number of ladies and gentlemen present evinced the great interest which is being taken by the citizens of Buffalo in the progress of a higher medical education. As many were unable to get admittance, it is to be hoped that another year the authorities of the University will hold convocation in a larger hall.

The absence in Europe of the Right Reverend Chancellor, the Bishop of Buffalo, was greatly deplored; in his place, the Vice-Chancellor, Rev. P. V. Kavanagh, presided; and, having declared convocation open, made an address, in which he briefly referred to the successful work being done by the several faculties of divinity, arts, medicine and law. At the close of his remarks, the successful candidates for the degree, clad in their academic gowns, were presented to the Vice-Chancellor by Dr. S. T. Clark, and, following the beautiful and impressive ritual employed by this University, they severally received the hood of the Doctor in Medicine, and subscribed to the following declaration in the University book:

"We, the graduates of the Medical Department of Niagara University, promise to cherish in our calling a feeling of deepest responsibility, recognizing therein the necessity for the continued study and cultivation of the science and art of medicine and surgery in sincerity and truth, by which shall be shown forth in our lives the duties and moral obligations devolving upon us, as members of a noble profession, and as scholars of a school whose teachings shall ever be our guide to that onward course in her motto—'*Religio, Mores, Cultura.*'"

The names of the gentlemen who have thus worthily won admittance into the profession, are as follows:

Henry Clark Buswell, Buffalo, N. Y.; John Joseph Twohey, Buffalo, N. Y.; Howard Lincoln Hunt, Hamburgh, N. Y.; John Francis Mulherin, Syracuse, N. Y.; Edward Nicholas Pfohl, Buffalo, N. Y.; Westervelt Banta, Buffalo, N. Y.; John Joseph Finerty, Honesdale, Pa.; George Hervey McMichael, Brantford, Ont.; Eugene Edward Martin, Buffalo, N. Y.; Clarence Butrick Le Van, Middleport, N. Y.; Sydney Algernon Dunham, Warren Center, Pa.; Anthony Schoonhart, Buffalo, N. Y.

The address to the graduates was delivered by Dr. Henry Flood, of Elmira. It appears in full in the pages of the JOURNAL, and we commend it to the perusal of all our readers. We regret that we cannot also publish in full the admirable address of Rev. James T. Edwards, A. M., D. D., president of Chamberlain Institute at Randolph. He spoke for nearly an hour, greatly delighting his hearers with his charming eloquence and his effective presentation and illustration of his subject.

He began by saying that in his own mind the prototype of the doctor was he who bravely takes his life-boat and goes out upon the tempestuous sea to rescue shipwrecked lives. He vividly compared the earnestness, zeal and self sacrifice of the physician with those of the life-saver. At times the physician stands forth in the view of the whole world. Every nation has anxiously watched the course of Dr. Mackenzie, the medical attendant of the Crown Prince, knowing full well that the fate of empires hangs upon his skill. The whole American nation was in sympathy with those doctors in New York who had attacked the enemy which had broken into the citadel of the mind of Roscoe Conkling. The doctor's work is a high trust. He is admitted into the very penetralia of the family, and becomes the repository of their well-guarded secrets. He should have a nice sense of honor. The world has never ceased to feel a deep contempt for the doctor who removed the drapery from the lifeless form of Lord Byron, and, discovering his club foot, proclaimed to the public the existence of a deformity which its possessor had so carefully concealed. Courtesy and sympathy are qualities which make the physician the more popular, even if they add nothing to his skill. It is well to cultivate that sympathy which ceases to be an emotion, but becomes a motive. The young doctor should avoid jumping at conclusions, and should not fall into the errors of phrenology, faith or mind cure and materialism. The address most happily set forth the duties and responsibilities of the medical man. The above sentiments indicate only the drift of the speaker's thought, but no extracts can do justice to the eloquent address.

At the close of convocation, the Faculty and invited guests assembled at "The Genesee," where over 100 sat down to enjoy the good cheer furnished at the alumni banquet. The menu was worthy the reputation of "The Genesee." The first toast of the evening was in honor of Dr. John Cronyn, president of the Faculty, whose absence through serious sickness would have cast a gloom over the assemblage were it not for the joyful knowledge that he was safely convalescent.

We have not space to note the many admirable responses to the various succeeding toasts. Pre-eminent among all, however, was the response of Prof. F. R. Campbell to the toast of the Medical Department of the University. This, written in heroic verse, sparkled with wit, humor and satire, and the successive "hits" at the special pursuits, peculiarities and vagaries of individual professors, caused much laughter and amusement.

